# Fat Deposition in the Muscle of Female and Male Yak and the Correlation of Yak Meat Quality with Fat

**DOI:** 10.3390/ani11072142

**Published:** 2021-07-20

**Authors:** Lin Xiong, Jie Pei, Min Chu, Xiaoyun Wu, Qudratullah Kalwar, Ping Yan, Xian Guo

**Affiliations:** 1 Animal Science Department, Lanzhou Institute of Husbandry and Pharmaceutical Sciences, Chinese Academy of Agricultural Sciences, Lanzhou 730050, China; xionglin@caas.cn (L.X.); peijie@caas.cn (J.P.); chumin@caas.cn (M.C.); wuxiaoyun@caas.cn (X.W.); 2 Key Laboratory for Yak Genetics, Breeding, and Reproduction Engineering of Gansu Province, Lanzhou 730050, China; 3 Department of Animal Reproduction, Shaheed Benazir Bhutto University of Veterinary and Animal Sciences, Sakrand 67210, Pakistan; qudratullahkalwar@gmail.com

**Keywords:** yak, fat, PPAR signaling, meat quality, gender

## Abstract

**Simple Summary:**

The type and quantity of fat are among the key factors influencing the carcass and meat quality in yak. The effect of gender to fat in yak and the correlation of meat quality with fat were studied in this paper. The meat of male yaks (MYs) is healthier from the point of nutritional value, whereas the meat of female yaks (FYs) possesses better qualities in relation to the visual impact and mouthfeel. The below results can provide a theoretical basis for the deep processing of yak meat. Yak meat quality (especially tenderness) can be improved by increasing the content of fat, C18:0, *cis*-C18:2, and *cis*-C18:1 in muscle during yak production. These genes, including *SCD, PLIN5, LPL, ME1* and *DBI*, play a crucial role in the regulation of fat deposition in yak. Above candidate genes for fat deposition in yak can supply the theoretical basis in molecular breeding of yak with a high fat content in muscle.

**Abstract:**

This study aimed to explore the differences in fat deposition between female (FYs) and male yaks (MYs). Compared with MYs, the tenderness, L*, marbling, absolute content of fat, and most fatty acids (FAs) of *longissimus dorsi* (LD) in FYs were higher or better (*p* < 0.05), whereas the relative content of polyunsaturated fatty acids (PUFAs) and n-3 PUFAs were lower (*p* < 0.01). The absolute content of fat, C18:0, *cis*-C18:2, *cis*-C18:1, and C24:0 were positively correlated with L*_45 min_, b*_24 h_, tenderness, and marbling score of LD in FYs and MYs (*p* < 0.05), respectively. *LPL*, *FATP2*, *ELOVL6*, *HADH*, *HACD*, and *PLINS* genes play a crucial role in improving the marbling score and tenderness of yak meat. The results of gene expression and protein synthesis showed the effect of gender to FA biosynthesis, FA transport, lipolysis, and FA oxidation in the adipose tissue of yak was realized by the expressions of ME1, SCD, ACSL5, LPL, FABP1, PLIN4, and PLIN2 in peroxisome proliferators-activated receptor (PPAR) signaling. This study established a theoretical basis for the improvement of the meat quality of yak and molecular breeding.

## 1. Introduction

Fat and fatty acids (FAs) composition are closely related to meat quality, including the appearance, texture, flavor, and hardness [1,2,3,4], and have an essential part in avoiding cold shortening, drip loss, and dark cutting [5]. Optimal intramuscular fat is crucial in animal husbandry [6,7]. Along with the rapid development of globalization and the change of consumer philosophy, the demand, supply, and acceptance of unconventional and exotic meats are increasing around the world [8,9,10,11]. Yak (*Bos grunniens*) is a unique livestock in Tibetan Plateau and its adjacent areas at heights of 2500–6000 m above sea level [12] possess unusual physiological adaptations to low temperature (as low as −40 °C) and full-grazing style with grasses, sedges, and fobs as their sole nutritional source [13]. At present, there are approximately 14 million yaks in the world. Yak meat is a unconventional meat, and the major source of animal protein in the local human diet [14,15]. Because of its rich protein and low fat, devoid of anabolic steroids and pollutants, yak meat is increasingly popular with consumers [16]. The annual yak meat yield is more than 500 thousand tons. The carcass yield and dressing percentage of yak are 46.67–51.0% and 37.10–43.02%, respectively. Because of special natural environment and traditional view, the yak breeding model is mainly natural grazing, and the slaughtering industry of yak is scattered. For a long time, the yak industry is an extensive management and low production performance. There are very few modern yak meat processing facilities in the place of origin. Yak meat classification and grade are not put to wide use in the industry, and the deep processing of yak meat is still in the initial stage.

Genetic factors [17], diet [18], gender [19], breeding environment [20,21], and so on can influence the FA profiles in animal-derived food. The metabolic responses are different in female and male animals [22,23], and the effect of gender on fat is realized by hormones [24]. It is very difficult to explore the regulation of fat deposition by a single technique, and the introduction of omics technologies has made these analyses possible. The high-throughput profiling of transcripts has been applied in the study on fat in cattle [25,26] and pig [27,28]. The newest RNA-sequencing (RNA-Seq) analysis can be used to examine the mRNA level in the adipose tissue of yaks with different genders to estimate its gene expression profile. The protein is the ultimate bio-function executor, and the proteomic analysis may provide more direct information on key biological processes. The emerging technology of quantitative proteomics has also been applied in the systematic study of changes in proteome-wide expression profiling in fat of cattle [29] and pig [30].

The goals of the research work was to investigate the regulative mechanism of gender to fat deposition in yak and the correlation of yak meat quality with fat in the muscle of yak. The fat content, FA profiles, and meat quality in *longissimus dorsi* (LD) of female yaks (FYs) and male yaks (MYs) were determined. In addition, the correlations of fat and FAs with meat quality were analyzed. Finally, the effect of gender on fat deposition in yaks was explored by transcriptome and proteome data in the adipose tissue. Differentially expressed genes (DEGs) and proteins (DEPs) in the adipose tissue of FYs and MYs were identified and annotated by Gene Ontology (GO) and the Kyoto Encyclopedia of Genes and Genome (KEGG). This research can establish a theoretical basis for the improvement of the yak meat quality and molecular breeding, and promote the development of yak breeding and deep processing of yak meat.

## 2. Materials and Methods

### 2.1. Animals and Samples Collection

An animal experiment was carried out in the pasture in Haiyan County, Qinghai province, China. Six FYs (four-years-old) and six MYs (four-years-old) were kept in grazing conditions, and were given free-choice access to diet and water. The contents of ash, crud fat, crud protein, neutral detergent fiber, acid detergent fiber, calcium, and phosphorus in grass in September were 7.66 ± 0.06%, 2.63 ± 0.20%, 11.93 ± 0.40%, 76.14 ± 0.79%, 10.09 ± 0.62%, 5.22 ± 0.32%, and 0.07 ± 0.002%, respectively. The intramuscular fat content in yak is so low that it is impossible to obtain an intramuscular fat sample for the transcriptome and proteome analysis; moreover, when the LD samples were directly used in metabolome and proteome analysis, the pre-tests showed that only minimal information on fat in yaks was obtained. Both physiological characteristics and form of the adipose tissue on the surface of the LD are similar to intramuscular fat, and are also easily collected. Thus, the adipose tissue on the surface of LD (12th–13th rib level) of the yak was chosen and used in this study. By late September, all yaks were humanely slaughtered at a commercial abattoir, and the samples of LD (12–13th rib level, 1000 g) and adipose tissue (10 g) were collected. The adipose tissue samples were placed in enzyme-free cryopreservation tubes and stored in liquid nitrogen; a part of LD samples were frozen at −20 °C for fat and FAs analysis, and the other part of LD samples were used for meat quality analysis.

### 2.2. Meat Quality Measurements

After evisceration and cleaning, carcasses were kept in cold storage (4 °C) until rigor mortis completion. The LD sample was used for an ageing period of one day (24 h post-mortem) and for the analysis of color, pH, cooking holding percentage, marbling score, and shear force. Meat color was measured with a CR-400 chroma meter (KONICA MINOLTA Inc., Tokyo, Japan) at 45 min and 24 h postmortem at 4 °C after slaughter, respectively, and the results were showed using International Commission on illumination (CIE) standards for lightness (L*), redness (a*), and yellowness (b*). The pH was measured using the TESTO 205 pH meter (TESTO AG Inc., Lenzkirch, Germany) at 45 min and 24 h at 4 °C after slaughter, respectively. The pH meter was calibrated using pH = 4 and 7 standard solutions from Mettler Toledo (Zurich, Switzerland). Shear force was measured using the C-LM4 tenderness meter (Northeast Agricultural University, Shenyang, China). The LD sample was boiled in a water bath at 80 °C until the core temperature of meat was 70 °C, and then cooled to 4 °C. Three replicate blocks were cut parallel to the side of the muscle fibers from each sample and each block sheared in the center in the vertical direction to the longitudinal orientation of the fibers. Shear force was the average peak positive force value for all individual sample blocks. The marbling score was visually determined with the aid of standard United States Department of Agriculture (USDA) cards. The LD sample was cut open 24 h post-mortem, and the marbling score was evaluated by the figures of grading standards. Cooking holding percentage was expressed as the weight change percentage of the sample before and after cooking according to the method in the China national standard NY/T821–2019 [31], and was measured by weighing after boiling the sample in the water bath at 80 °C.

### 2.3. Determination of Fat Content and Fatty Acid (FA) Profiles

The fat content in LD was determined according to the Soxhlet extraction principle using a Soxtec 2050 soxhlet apparatus (FOSS Inc., Hillerød, Denmark). FA profiles in LD were analyzed according to the method described in Song et al. [32], and a total of 37 FAs were determined in this study. First, the fat in LD was extracted with the solution of chloroform and methanol (*v*:*v*, 2:1) for three times, and the combined extract solution was dried under nitrogen blow. Next, the extracted fat sample was decomposed into non-esterified FAs by the basic hydrolysis. Finally, the non-esterified FAs were derived with boron fluoride-methanol solution. The 7890A gas chromatography system (Agilent Corp., Santa Clara, CA, USA) coupled with a flame ionization detector and an Agilent J&WCP-Sil88 FAME capillary column (100 m × 0.25 mm × 0.20 μm) were used to separate and detect the content of fatty acid methyl esters (FAMEs). The nitrogen constant linear flow rate was at 0.5 mL/min, and the split ratio was 1:100. The initial column temperature was held at 100 °C for 5 min, increased to 180 °C at 8 °C/min, and held for 9 min. Then, the temperature was increased to 230 °C at 1 °C/min and held for 15 min. The injector and detector temperature were at 260 and 280 °C, respectively. The injection volume was 1 μL. The analytes were determined based on their retention times, and FAs contents were calculated by FAMEs.

### 2.4. Transcriptome Analyses

Three fat samples were randomly chosen in each group. Total RNA was extracted using the mirVana^TM^ miRNA Isolation Kit (Ambion Inc., Foster City, CA, USA). The purity of RNA was determined by evaluating absorbance using a NanoDrop 2000 spectrophotometer (Thermo Fisher Scientific, Santa Clara, CA, USA). RNA integrity was evaluated using the Agilent 2100 Bioanalyzer (Agilent Technologies, Santa Clara, CA, USA). The mRNA libraries for sequencing were prepared using TruSeq Stranded mRNA LTSample Prep Kit (Illumina, San Diego, CA, USA). Then, these libraries were sequenced on the HiSeqTM 2500 sequencing platform (Illumina, San Diego, CA, USA).

### 2.5. Protein Preparation and Digestion

The fat samples for transcriptome analyses were adopted for proteome analysis too. Frozen fat samples were lysed, followed by sonication and centrifugation at 12,000 r/min twice. The sample containing 100 μg protein was diluted with reducing buffe in an ultrafiltration tube and the dithiothreitol solution was added in, following by incubation at 55 °C for 30 and cooling to room temperature. The iodoacetamide solution was added into the tube, and the solution was shaken well and mix stood for 15 min. Acetone was then added to precipitate protein and leaving for over 4 h at −20 °C. Then, the solution was centrifuged and the sediment was redissolved with TEAB2, followed with sequencing-grade trypsin in each tube. The solution was incubated for digestion at 37 °C, and centrifuged and lyophilized. Eighty-eight μL acetonitrile was added into the tandem mass tag (TMT) reagent vial at room temperature, followed by vortex and centrifugation. Then, 41 μL TMT label reagent was added into the sample, and the tube was incubated for 1 h at room temperature. Finally, the reaction was terminated with hydroxylamine.

### 2.6. Reversed-Phase Liquid Chromatography (RPLC) Separation and Mass Spectrum (MS) Analysis

Reversed-phase liquid chromatography (RPLC) separation was performed on a 1100 HPLC System (Agilent Technologies, Santa Clara, CA, USA) using an Agilent Zorbax Extend RP column (5 μm, 150 mm × 2.1 mm). The mobile phases were A (2% acetonitrile in HPLC water) and B (98% acetonitrile in HPLC water), and the flow rate was 300 μL/min. The detection wavelengths were at 210 and 280 nm, respectively. The sample was collected over 8–60 min, and the separated peptides were lyophilized for mass spectrometry (MS). MS analyses were performed by a Q-Exactive mass spectrometer (Thermo Fisher Scientific, Waltham, MA, USA), equipped with a Nanospray Flex source (Thermo Fisher Scientific, Waltham, MA, USA). The sample was separated by a C_18_ column (15 cm × 75 µm). The mobile phases were A (0.1% formic acid in water) and B (80% acetonitrile, in water containing 0.1% formic acid), and the flow rate was 300 nL/min and linear gradient was 0–40 min, 5–30% B; 40–54 min, 30–50% B; 54–55 min, 50–100% B; 55–60 min, 100% B. Full MS scans were acquired in the mass range of 300–1600 m/z with a mass resolution of 70,000.

### 2.7. Statistical Analysis

Fat content, FAs content, a*, b*, L*, pH, shear force, marbling score, and cooking holding percentage were calculated separately using independent-sample T test in SPSS 16.0, and the correlations of meat quality with fat and FAs were calculated separately using Pearson correlation analysis in SPSS 16.0 too. The numbers of reads in the RNA-seq analysis were normalized against reads per kilobase of transcripts per million to compute the gene expression levels. Fragments per kilobase of transcript per million mapped reads (FPKM) of each gene were calculated using cufflinks, and the read counts of each gene were obtained by htseq-count. DEGs were identified using the DESeq (2012) R package functions estimate SizeFactors and nbinomTest. Proteome Discoverer (v. 2.3) was used to search all of the Q Exactive raw data thoroughly against the sample protein database. A database search was performed with Trypsin digestion specificity. Alkylation on cysteine was considered as a fixed modification in the database searching. A global false discovery rate (FDR) was set to 0.01, and protein groups considered for quantification were required at least two peptides. In this study, *p* < 0.05 and Fold Change (FC) > 2 or FC < 0.5 were set as the threshold for DEGs and DEPs. The transcriptome and proteome data can reveal the molecular regulation of gender to fat deposition in yak, and the Pearson correlation analysis can explore the main factors on fat which can improve the meat quality in yak.

## 3. Results

### 3.1. Meat Quality 

The results of meat quality of LD in FYs and MYs are shown in Table 1. Both L*_45 min_ and L*_24 h_ were higher in FYs vs. MYs (*p* < 0.05), and b*_24 h_ was higher in FYs vs. MYs (*p* < 0.05). The shear force was lower in FYs vs. MYs (*p* < 0.01). Moreover, the marbling score was higher in FYs vs. MYs (*p* < 0.05). Meanwhile, no significant differences in other indicators of meat quality were found between FYs and MYs.

### 3.2. Fat Content and FA Profiles 

Fat contents in LD of FYs and MYs were 2.51 ± 0.10% and 1.83 ± 0.09% (*p* < 0.01), respectively. The absolute and relative contents of FAs in LD of FYs and MYs are shown in Table 2. A total of 34 FAs were simultaneously detected in LD of FYs and MYs, including 17 saturated fatty acids (SFAs), 8 monounsaturated fatty acids (MUFAs), and 9 polyunsaturated fatty acids (PUFAs). The absolute contents of 16 FAs in LD were higher in FYs vs. MY (*p* < 0.05), whereas 10 FAs in LD were lower in FYs vs. MY (*p* < 0.01). The absolute contents of ΣSFAs, total unsaturated fatty acids (ΣUFAs), ΣMUFAs, and ΣPUFAs in LD were all higher in FYs vs. MYs (*p* < 0.01). The FAs in LD of both FYs and MYs largely consisted of C18:0, *cis*-C18:1, *cis*-C18:2, C16:0, C20:4n6, C24:0, C24:1, and *cis*-C20:5n3. On the other hand, the relative content of ΣSFAs in LD was higher in FYs vs. MYs (*p* < 0.01), whereas the relative content of ΣUFAs was lower (*p* < 0.01). Further, the relative content of ΣPUFAs in LD was lower in FYs vs. MY (*p* < 0.01), whereas the relative content of ΣMUFAs was higher in FYs vs. MY (*p* < 0.01).

The relative contents of Σn-3 and Σn-6 PUFAs, and the ratio of different types of FAs in LD of FYs and MYs are shown in Table 3. The ΣPUFAs/ΣSFAs (relative content) in LD of FYs and MYs were 0.38 and 0.50 (*p* < 0.01), respectively, and the relative content of Σn-3 and Σn-6 PUFAs in LD of FYs and MYs were 2.15 and 16.22%, and 6.65 and 17.46% (*p* < 0.05), respectively. Meanwhile, the Σn-6/Σn-3 PUFAs in LD of FYs and MYs were 8.53 and 2.44 (*p* < 0.01), respectively.

### 3.3. Function Enrichment Analysis for Differentially Expressed Genes (DEGs) and Differentially Expressed Proteins (DEPs)

**Figure 1:**
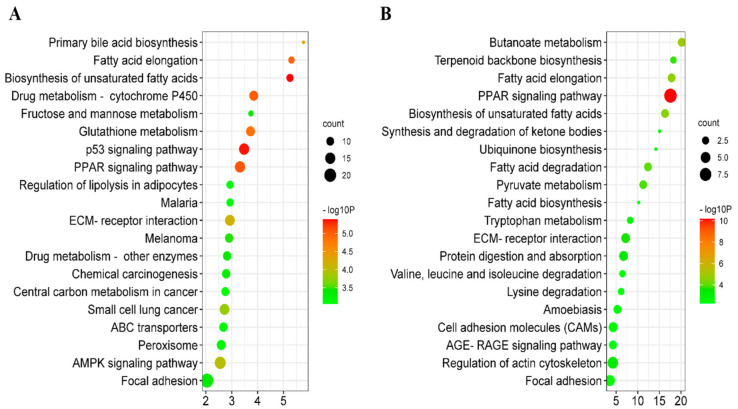
(**A**) The bubble diagram of the top 20 Kyoto Encyclopedia of Genes and Genomes (KEGG) enrichment pathways of differentially expressed genes (DEGs). The horizontal axis represents the enrichment score; (**B**) The bubble diagram of the top 20 KEGG enrichment pathways of differentially expressed proteins (DEPs).

There were 1027 DEGs in adipose tissue in FYs vs. MYs and 611 genes were upregulated, whereas 416 genes were downregulated. Moreover, there were 82 DEPs in the adipose tissue in FYs vs. MYs, and 53 DEPs were upregulated, whereas 29 DEPs were downregulated. The biological processes of GO enrichment of DEGs were mostly involved in long-chain FA transport (GO:0015909), FA β-oxidation (GO:0006635), fructose 2,6-bisphosphate metabolic process (GO:0006003), extracellular polysaccharide biosynthetic process (GO:0045226), bicarbonate transport (GO:0015701), cell adhesion (GO:0007155), and renal water absorption (GO:0070295). The biological processes of GO enrichment of DEPs were mostly involved in collagen fibril organization (GO:0030199), collagen biosynthetic process (GO:0032964), acute-phase response (GO:0006953), positive regulation of cell division (GO:0051781), UFA biosynthetic process (GO:0006636), and very long-chain FA biosynthetic process (GO:0042761). Total 455 DEGs were enriched in 65 KEGG pathways (*p* < 0.05) which were mainly involved in various substance metabolisms and signaling pathways (Figure 1A). Moreover, a total of 82 DEPs were enriched in 52 KEGG pathways (*p* < 0.05) (Figure 1B). The mutual pathways on KEGG enrichment of DEGs and DEPs included the biosynthesis of unsaturated fatty acids (bom01040), fatty acid elongation (bom00062), terpenoid backbone biosynthesis (bom00900), butanoate metabolism (bom00650), fatty acid degradation (bom00071), tryptophan metabolism (bom00380), synthesis and degradation of ketone bodies (bom00072), and PPAR signaling pathway (bom03320). The important DEGs and DEPs in the above crucial KEGG pathways are shown in Table 4.

**Figure 2:**
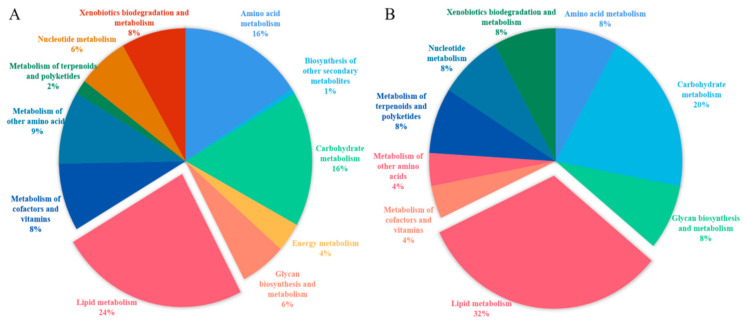
(**A**) The distribution of DEGs in the metabolism classification at KEGG level2; (**B**) The distribution of DEPs in the metabolism classification at KEGG level2.

Further, DEGs distribution in the metabolism classification at KEGG level2 showed (Figure 2A) that a total of 41 DEGs were involved in lipid metabolism and their percentage in all DEGs was 24%, which was the largest. Meanwhile, the percentage of DEGs in carbohydrate metabolism (16%) and amino acid metabolism (16%) were larger too. On the other hand, DEPs were largely concentrated in lipid metabolism (32%), carbohydrate metabolism (20%), and amino acid metabolism (8%) in the metabolism classification at KEGG level2 (Figure 2B) too. A total of eight DEPs were involved in lipid metabolism, including ACAT2, HACD3, LPL, SRD5A3, ACSL5, HADH, SCD, and ELOVL6, and they play a crucial role in fatty acid elongation, fatty transport, and fatty acids synthesis, and their expressions were all upregulated in the adipose tissue of FYs (*p* < 0.05).

## 4. Discussion

### 4.1. Correlation of Fat with Meat Quality

Consumers much prefer the beef which is low L* and b* and high a* with abundant marbling and better tenderness, and when the beef possessing above quality is higher price. In this study, the L*, tenderness, and marbling score of LD in FYs were higher than that in MYs, and the FYs meat quality is better from the view of visual impact and mouthfeel. Meat color may also be influenced by factors such as diet, meat pH, animal age, and intramuscular fat content [33]. In this study, most of these factors, except intramuscular fat content, is kept the same, and the intramuscular fat content in yaks is closely related to meat color. An increase in ΣSFAs (absolute content) in LD of FYs mainly resulted from C16:0, C18:0, and C24:0, whereas cis-C18:1, C24:1, cis-C18:2n6, and C20:4n6 were responsible for the decrease in ΣPUFAs (absolute content). These results showed that the degradation of long-chain PUFA may contribute to the increase in short-chain SFAs [34]. C18:0 has a neutral effect on blood cholesterol, and exerts a neutral effect like some MUFAs [35]. The FAs that appears in greater proportion is C18:0, which is a nutritionally positive aspect of yak intramuscular fat. Generally speaking, the C18:0 is higher in bulls compared to cows [36], and the above result is also suitable for yak. The relative content of FAs is crucial when evaluating the meat quality [37,38]. The Food and Agriculture Organization of United Nations (FAO) and the World Health Organization (WHO) suggest a reduction in the intake of SFAs and trans fatty acids (TFAs), and an increase in the intake of n-3 PUFAs [39]. The recommendation of ΣPUFAs/ΣSFAs is above 0.4, while Σn-6/Σn-3 PUFAs is below 4. Two TFAs, including *trans*-C18:2n6 and *trans*-C18:1, were determined in LD of both FYs and MYs, and the absolute content of *trans*-C18:1 was higher in LD of FYs (*p* < 0.01), but no differences in their relative contents were found; the ΣPUFA/ΣSFA in beef is typically 0.1 [40]. The low fat content in LD of yaks explains the ΣPUFA/ΣSFA values, which are higher than the typical values for beef. In addition, under grazing conditions, Σn-3 PUFAs concentrations of yak meat were higher than that of beef cattle meat [41], and the yak meat showed more favorable Σn-6/Σn-3 PUFAs than reported for beef. The ΣPUFAs/ΣSFAs (relative contents) in LD of MYs was above the recommended levels, while the Σn-6/Σn-3 PUFA (relative contents) was below the recommended levels. Therefore, from the view of fat nutritional value, yak meat is healthier than usual beef, and MYs meat is healthier than FYs meat.

The results of Pearson correlation analysis between meat quality and fat content in FAs (absolute content) are shown in Table 5. There were negative correlations between fat content and shear force in LD of FYs and MYs (*p* < 0.05). There were positive correlations between the fat content and the L*_24 h_, marbling score in LD of MYs (*p* < 0.05), and the fat content and L*_24 h_, b*_24 h_, marbling score in LD of FYs (*p* < 0.05). The absolute contents of C18:0, *cis*-C18:1, and C24:0 in LD of the MYs were positively correlated with the L*_24 h_ and marbling (*p* < 0.05), respectively, whereas they were negatively correlated with shear force (*p* < 0.05). The absolute contents of C18:0 and *cis*-C18:2 in LD of the FYs were positively correlated with L*_45 min_, b*_24 h_, marbling score (*p* < 0.05), respectively, whereas they were negatively correlated with shear force (*p* < 0.05) too. Therefore, a higher fat content can improve the tenderness of yak meat, and higher contents of C18:0, *cis*-C18:2 *cis*-C18:1, and C24:0 in yak meat can play a crucial role in improving b*, L*, marbling, and tenderness. Marbling score and tenderness are regulated by multiple intramuscular fat metabolic genes. The critical lipid uptake in genes, such as *LPL* and *FATP2*, are implicated in the process of fatty acid flux into adipocytes clustered along myofiber fasciculi in the muscle [42]. *ELOVL6*, *HADH*, *ACOT7,* and *HACD* genes are responsible for de novo synthesis of fatty acids, and act as key regulatory molecules in fat deposition. The *PLINS* gene is involved in the regulation of fat storage. The marbling score and tenderness in LD of FYs were higher, likely because of the overexpression of the above lipogenic-related genes. Further, the overexpression of *LPL*, *FATP2*, *ELOVL6*, *HADH*, *HACD*, and *PLINS* proteins verified the regulation of above genes to the marbling score and tenderness in yaks with a different gender.

### 4.2. Effect of Gender to Fat Deposition in Yak

The absolute contents of ΣSFAs, ΣUFAs, ΣPUFAs, and ΣMUFAs in LD of FYs were higher (*p* < 0.01), and the capacity of fat deposition in FYs is more powerful than in MYs. Both DEGs and DEPs in the adipose tissue of FYs and MYs were largely concentrated in lipid metabolism, carbohydrate metabolism, and amino acid metabolism in the metabolism classification at KEGG level2, and the fat deposition in yaks with different gender was determined by the synergism of fat, amino acid, and carbohydrate metabolism. Amino acid can transfer into acetyl-Coa by butanoate metabolism (bom00650) (*p* < 0.05), and the expression of DEGs (*MAOB*, *AADAT*, *HADH*, *ACMSD,* and *ACAT2*) and DEPs (HADH and ACAT2) in tryptophan metabolism (bom00038) (*p* < 0.05) were all upregulated (*p* < 0.05) in the adipose tissue of FYs. Therefore, more amino acids, especially tryptophan in the adipose tissue of FYs, were converted into acetyl-CoA, and further acetyl-CoA was converted into triglyceride. On the other hand, the conversion from carbohydrate into triglyceride can be realized by citric acid-pyruvate cycle, and the expression of DEGs (*MAOB*, *AADAT*, *HADH*, *ACMSD* and *ACAT2*) and DEPs (ACAT2 and ME1) in pyruvate metabolism (bom00620) (*p* < 0.05) were all upregulated (*p* < 0.05) in the adipose tissue of FYs, and more carbohydrates in the adipose tissue of FYs were converted into triglyceride.

**Figure 3:**
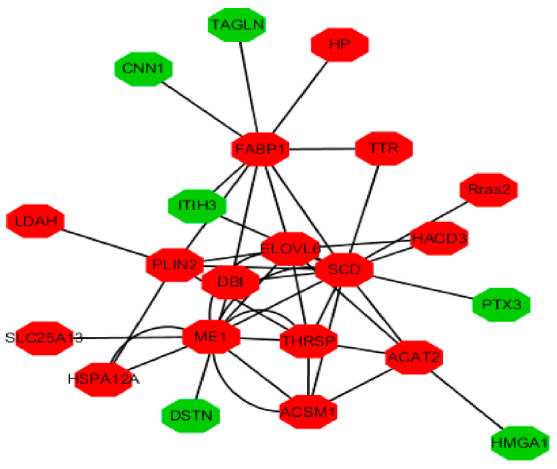
The interaction networks of DEPs related to fat metabolism in the adipose tissue of FYs and MYs. The red expressed the upregulated expression of protein in the adipose tissue of FY, and the green expressed the downregulated expression of protein in the adipose tissue of FY.

The fat deposition is most directly determined by fat metabolism, and FAs synthesis is crucial for fat synthesis and fat deposition. The interaction networks of DEPs related to fat metabolism in the adipose tissue of FYs and MYs are shown in Figure 3. The FA elongation mainly synthesizes the SFAs. The expressions of DEGs (*HADHB* [43], *HADH*, *ELOVL6* [44], *HSD17B8* [45], *HACD2* [46], and *ACOT7* [47]) and DEPs (ELOVL6, HADH, and HACD3) in fatty acid elongation (bom00062) (*p* < 0.05) were all upregulated in the adipose tissue of FYs (*P_FD_*_R_ < 0.05); these genes can positively regulate the SFAs biosynthesis. The absolute contents of ΣSFAs, C4:0, C6:0, C10:0, C13:0, C14:0, C16:0, C18:0, C21:0, and C24:0 in LD of FYs were higher than that in LD of FYs. The expressions of DEGs (*SCD* [48], *ACOT7*, *HACD2*, *SCP2* [49], *ELOVL6*, *ACAA1* [50], and *HSD17B12*) and DEPs (SCD, ELOVL6, and HACD3) in UFAs biosynthesis (bom01040) (*p* < 0.05) were all upregulated in the adipose tissue of FYs (*p* < 0.05), and they can positively regulate the UFAs biosynthesis, especially n-6 PUFAs (C18:2n6). SCD is the first enzyme converting SFA to MUFA and PUFA. More SFAs were converted to MUFAs in the adipose tissue of FYs. The absolute contents of ΣMUFAs, ΣPUFAs, Σn-6 PUFAs, cis-C18:2n6, and C20:4n6 in LD of FYs were higher. Therefore, both SFAs and UFAs synthesis in the adipose tissue of FYs increased, which resulted in the fat content in LD of FYs being higher than that in LD of MYs. The correlations of fat with L*_24h_, marbling score, and tenderness were positive, so higher fat content can improve the meat quality of yak.

**Figure 4:**
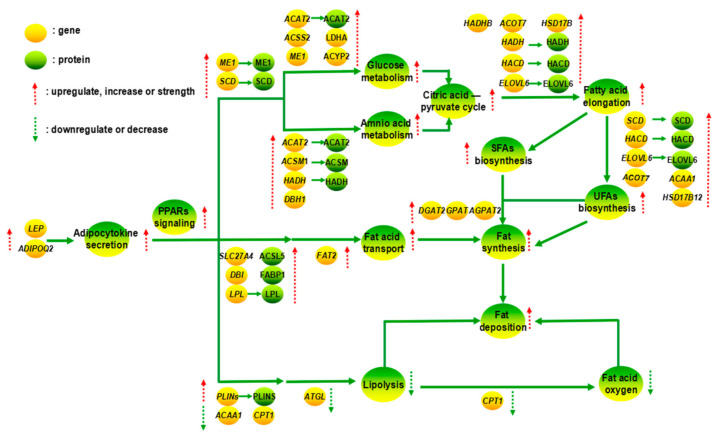
The effect of different gender to fat metabolism in yaks by PPAR signaling pathways.

PPAR, members of the nuclear hormone receptor super family, can influence gene transcription of fatty metabolism enzymes. Leptin (LEP) acts as an appetite-regulating factor and is an essential factor in fat deposition [51,52]. Moreover, it can activate the PPAR signaling. *LEP* and *ADIPOQ2* genes are responsible for adipocyte secretion, and the expressions of *LEP* and *ADIPOQ2* genes were upregulated in the adipose tissue of FYs (*P_FDR_* < 0.05). The enrichment scores of PPAR signaling pathway (bom03320) on KEGG enrichment of DEGs and DEPs were 3.32 and 17.60, respectively, which were the highest values in the KEGG pathways regulating fat metabolism. Meanwhile, FA elongation and biosynthesis of UFAs can be contained in this pathway. The fat synthesis, lipolysis, fatty acid transport, and fatty acids oxygen were regulated by PPAR signaling in the adipose tissue of yaks with a different gender (Figure 4). The downstream DEGs in PPAR signaling pathway included *FABP*, *ME1*, *SCD*, *ACBP* [53], *LPL*, *CPT1*, *ACSL5* [54], *ACAA1*, and *PLIN5* [55], and DEPs included ACSL5, SCD, PLIN4, FABP1, PLIN2, LPL, ME1, and DBI (*p* < 0.01). Moreover, adipose triacylglycerol lipase (ATGL) is the central enzyme for fatty acid catabolism and is capable of hydrolyzing TG into diglycerides. Diacylglycerol O-acyltransferase 2 (DGAT2) is a core regulator of TG synthesis. The regulation of ATGL and DGAT2 to fat metabolism is achieved by the PPAR signaling.

## 5. Conclusions

The fat contents and the absolute content of ΣSFAs, ΣUFAs, ΣPUFAs, and ΣMUFAs in LD of FYs were all higher than that in MYs, whereas the relative content of ΣPUFAs and Σn-3 PUFAs in LD of FYs were lower. The FAs composition in MYs meat is healthier for consumers, whereas the tenderness, L*, and marbling score of LD of FYs are better from the viewpoint of vision. In addition, positive correlations were found between fat content and L*_24 h_, marbling, whereas negative correlations were found between fat contents and shear forces; the absolute contents of C18:0, *cis*-C18:2, *cis*-C18:1 and C24:0 were positively correlated with L*_45 min_, b*_24 h_, marbling score, respectively. The differences of the marbling score and tenderness in muscle of yaks with different gender is achieved by the regulation of *LPL*, *FATP2*, *ELOVL6*, *HADH*, *HACD*, and *PLINS* genes. Compared with MYs, the fat synthesis, FA biosynthesis, lipolysis, FA oxidation, and FA transport in yak with a different gender were regulated by ME1, SCD, ACSL5, LPL, FABP1, PLIN4, and PLIN2 in PPAR signal. The capacity of fat deposition in FYs was more powerful, and more fat was deposited in FYs.

## Figures and Tables

**Table 1:** The meat quality of *longissimus dorsi (LD)* in female (FYs) and male yaks (MYs).

Variable	FYs (Mean ± SE)	MYs (Mean ± SE)
a*_45 min_	55.03 ± 0.72	53.27 ± 0.47
b*_45 min_	23.34 ± 0.84	21.31 ± 1.09
L*_45 min_	22.77 ± 0.65 ^a^	19.83 ± 0.74 ^b^
a*_24 h_	55.78 ± 0.55	53.88 ± 0.59
b*_24 h_	24.49 ± 0.87 ^a^	21.41 ± 0.55 ^b^
L*_24 h_	23.98 ± 1.04 ^b^	20.82 ± 0.29 ^a^
pH_45 min_	5.86 ± 0.03	5.82 ± 0.03
pH_24 h_	5.40 ± 0.05	5.55 ± 0.06
Shear force (kg)	7.93 ± 0.16 ^A^	9.29 ± 0.27 ^B^
Marbling score	3.25 ± 0.16 ^a^	2.75 ± 0.10 ^b^
Cooking holding percentage (%)	70.60 ± 0.86	71.37 ± 0.36

SE: Standard Error. Values in the same row with different lowercase superscripts show *p* < 0.05, different capital superscripts show *p* < 0.01.

**Table 2:** The absolute and relative content of fatty acids (FAs) in LD of FYs and MYs.

FAs	Absolute Content (mg/100 g)		Relative Content (%)	
	FYs (Mean ± SE)	MYs (Mean ± SE)	FYs (Mean ± SE)	MYs (Mean ± SE)
ΣSFAs	1002.82 ± 14.94 ^A^	658.71 ± 15.74 ^B^	52.28 ± 0.33 ^A^	46.68 ± 0.43 ^B^
C4:0	3.46 ± 0.22 ^A^	2.22 ± 0.09 ^B^	0.18 ± 0.01	0.16 ± 0.01
C6:0	0.55 ± 0.04 ^A^	0.38 ± 0.01 ^B^	0.02 ± 0.001	0.03 ± 0.002
C8:0	0.12 ± 0.02 ^A^	0.55 ± 0.02 ^B^	0.01 ± 0.001 ^A^	0.04 ± 0.00 ^B^
C10:0	0.57 ± 0.03 ^a^	0.46 ± 0.02 ^b^	0.03 ± 0.001	0.03 ± 0.001
C11:0	0.09 ± 0.01	0.07 ± 0.003	0.004 ± 0.0003	0.005 ± 0.0002
C12:0	0.50 ± 0.04	0.39 ± 0.01	0.03 ± 0.002	0.03 ± 0.001
C13:0	1.25 ±0.13 ^a^	0.83 ± 0.03 ^b^	0.06 ± 0.01	0.06 ± 0.002
C14:0	9.86 ± 0.33 ^A^	8.03 ± 0.27 ^B^	0.515 ± 0.02	0.57 ± 0.02
C15:0	6.92 ± 0.56 ^A^	9.42 ± 0.30 ^B^	0.36 ± 0.03 ^A^	0.67 ± 0.02 ^B^
C16:0	271.66 ± 4.70 ^A^	181.69 ± 3.47 ^B^	14.18 ± 0.35 ^a^	12.88 ± 0.14 ^b^
C17:0	15.52 ± 1.79 ^a^	23.04 ± 1.44 ^b^	0.81 ± 0.08 ^A^	1.63 ± 0.08 ^B^
C18:0	581.96 ± 12.53 ^A^	341.49 ± 9.14 ^B^	30.33 ± 0.40 ^A^	24.20 ± 0.32 ^B^
C20:0	2.01 ± 0.25	1.60 ± 0.09	0.10 ± 0.01	0.11 ± 0.01
C21:0	6.48 ± 0.46 ^a^	4.95 ± 0.26 ^b^	0.34 ± 0.02	0.35 ± 0.01
C22:0	2.81 ± 0.19	2.34 ± 0.21	0.15 ± 0.01	0.17 ± 0.01
C23:0	11.66 ± 1.69	9.86 ± 0.28	0.61 ± 0.09	0.70 ± 0.02
C24:0	87.41 ± 4.92 ^a^	71.41 ± 3.33 ^b^	4.55 ± 0.21	5.06 ± 0.19
ΣMUFAs	536.21 ± 10.36 ^A^	423.15 ± 5.61 ^B^	27.95 ± 0.36 ^A^	30.02 ± 0.24 ^B^
C14:1	1.08 ± 0.11 ^A^	18.29 ± 1.22 ^B^	0.06 ± 0.01 ^A^	1.30 ± 0.08 ^B^
*cis*-C15:1	2.91 ±0.28 ^A^	5.10 ± 0.26 ^B^	0.15 ± 0.01 ^A^	0.36 ± 0.02 ^B^
C16:1	36.20 ± 3.59	37.01 ± 1.50	1.88 ± 0.17 ^A^	2.62 ± 0.10 ^B^
*cis*-C17:1	5.62 ± 0.70 ^A^	15.23 ±0.89 ^B^	0.29 ± 0.04 ^A^	1.08 ± 0.05 ^B^
*cis*-C18:1	424.98 ± 7.31 ^A^	300.62 ± 4.82 ^B^	22.16 ± 0.32	21.33 ± 0.26
*trans*-C18:1	11.24 ± 1.14 ^A^	6.08 ± 0.55 ^B^	0.59 ± 0.06	0.43 ± 0.04
*cis*-C20:1	4.97 ± 0.44 ^A^	1.79 ± 0.05 ^B^	0.26 ± 0.02 ^A^	0.13 ± 0.004 ^B^
C24:1	49.23 ± 3.07 ^a^	39.04 ± 1.04 ^b^	2.56 ± 0.14	2.77 ± 0.09
ΣPUFAs	379.29 ± 6.90 ^A^	328.71 ± 7.27 ^B^	19.77 ± 0.13 ^A^	23.30 ± 0.32 ^B^
*cis*-C18:2n6	274.54 ± 7.37 ^A^	173.92 ± 4.58 ^B^	14.31 ± 0.30	12.33 ± 0.23
*trans*-C18:2n6	0.53 ± 0.06	0.47 ± 0.04	0.03 ± 0.01	0.03 ± 0.01
*cis*-C18:3n6	5.33 ± 0.52 ^A^	10.71 ± 0.27 ^B^	0.28 ± 0.03 ^A^	0.76 ± 0.02 ^B^
C18:3n3	16.83 ± 2.48 ^A^	47.95 ± 2.28 ^B^	0.87 ± 0.12 ^A^	3.39 ± 0.11 ^B^
*cis*-C20:2	3.17 ± 0.50 ^A^	6.21 ± 0.32 ^B^	0.16 ± 0.02 ^A^	0.44 ± 0.02 ^B^
C20:4n6	54.56 ± 2.05 ^A^	43.61 ± 1.62 ^B^	2.84 ± 0.10	3.10 ± 0.13
*cis*-C20:3n3	0.25 ± 0.04	0.25 ± 0.01	0.01 ± 0.002	0.02 ± 0.001
*cis*-C20:5n3	21.57 ± 2.20 ^A^	43.28 ± 1.09 ^B^	1.13 ± 0.12 ^A^	3.07 ± 0.10 ^B^
*cis*-C22:6n3	2.51 ± 0.23	2.31 ± 0.07	0.13 ± 0.01 ^a^	0.16 ± 0.01 ^b^
ΣUFAs	915.50 ± 15.66 ^A^	751.86 ± 12.14 ^B^	47.72 ± 0.33 ^A^	53.32 ± 0.43 ^B^

ΣMUFAs: sum of monounsaturated fatty acids; ΣSFAs: sum of saturated fatty acids; ΣPUFAs: sum of polyunsaturated fatty acids; ΣUFAs: sum of unsaturated fatty acids. Values in the same row with different lowercase superscripts show *p* < 0.05, different capital superscripts show *p* < 0.01.

**Table 3:** The relative contents of Σn-3 and Σn-6 polyunsaturated fatty acids (PUFAs) and the ratio of different types of FAs in LD of FYs and MYs.

FAs	FYs (Mean ± SE)	MYs (Mean ± SE)
Σn-3 PUFAs (%)	2.15 ± 0.18 ^A^	6.65 ± 0.07 ^B^
Σn-6 PUFAs (%)	16.22 ± 0.21 ^a^	17.46 ± 0.34 ^b^
Σn-6/Σn-3 PUFAs	8.53 ±0.86 ^A^	2.44 ± 0.06 ^B^
ΣSFAs/ΣUFAs	1.10 ± 0.01 ^A^	0.88 ± 0.02 ^B^
ΣMUFAs/ΣPUFAs	1.41 ± 0.02 ^A^	1.29 ± 0.02 ^B^
ΣPUFAs/ΣSFAs	0.38 ± 0.01 ^A^	0.50 ± 0.01 ^B^

Σn-3 PUFAs: sum of n-3 polyunsaturated fatty acids; Σn-6 PUFAs: sum of n-6 polyunsaturated fatty acids. Values in the same row with different lowercase superscripts show *p* < 0.05, different capital superscripts show *p* < 0.01.

**Table 4:** The differentially expressed genes (DEGs) and differentially expressed proteins (DEPs) in the crucial Kyoto Encyclopedia of Genes and Genomes (KEGG) pathways related to fat metabolism in the adipose tissue of FYs and MYs.

KEGG Pathway	DEGs	DEPs
Biosynthesis of unsaturated fatty acids (bom01040)	*SCD*, *ACOT7*, *HACD2*, *SCP2*, *ELOVL6*, *HSD17B12*, *ACAA1*, *HSD17B12*	SCD, ELOVL6, HACD3
Fatty acid elongation (bom00062)	*ACOT7*, *HACD2*, *THEM4*, *HADHB*, *ELOVL6*, *HADH*, *HSD17B12*	ELOVL6, HADH, HACD3
Terpenoid backbone biosynthesis (bom00900)	*PCYOX1*, *ACAT2*, *FNTB*	PCYOX1, ACAT2
Butanoate metabolism (bom00650)	*ACSM1*, *HADH*, *BDH1*, *ACAT2*	ACSM1, HADH, ACAT2
Fatty acid degradation (bom00071)	*ACADSB*, *HADHB*, *HADH*, *ACAT2*, *ACAA1*, *CPT1C*	ACSL5, HADH, ACAT2
Tryptophan metabolism (bom00380)	*MAOB*, *AADAT*, *HADH*, *ACMSD*, *ACAT2*	HADH, ACAT2
Synthesis and degradation of ketone bodies (Bom00072)	*BDH1*, *ACAT2*	ACAT2
PPAR signaling pathway (bom03320)	*SCD*, *SCP2*, *PLIN5*, *ACOX2*, *ACAA1*, *LPL*, *ME1*, *CPT1C*, *SLC27A4*, *SLC27A6*, *DBI*, *SCP2*	ACSL5, SCD, PLIN4, FABP1, PLIN2, LPL, ME1, DBI
Pyruvate metabolism (bom00620)	*ACAT2*, *ACSS2*, *LDHA*, *ME1*, *SCP2*, *ACYP2*	ACAT2, ME1

**Table 5:** The Pearson correlation analysis between meat quality and FAs, fat content (absolute content) in LD of FYs and MYs.

Gender	Variable	L*_45 min_	b*_24 h_	L*_24 h_	Shear Force	Marbing
MYs	Fat content	0.41	0.79	0.89 ^a,b^	−0.84 ^a,b^	0.85 ^a,b^
	C18:0	0.43	0.87 ^a,b^	0.85 ^a,b^	−0.92 ^A,B^	0.76
	*cis*-C18:1	0.39	0.78	0.90 ^a,b^	−0.81 ^a,b^	0.91 ^a,b^
	*cis*-C18:2	0.15	0.31	0.44	−0.32	0.39
	C16:0	0.42	0.58	0.52	−0.61	0.35
	C20:4n6	0.23	−0.25	−0.21	−0.30	−0.28
	C24:0	0.29	0.77	0.85 ^a,b^	−0.81 ^a,b^	0.88 ^a,b^
	C24:1	−0.27	−0.55	−0.28	−0.56	−0.17
	*cis*-C20:5n3	0.56	−0.06	0.01	−0.12	−0.07
FYs	Fat content	0.94 ^A,B^	0.88 ^a,b^	0.93 ^A,B^	−0.84 ^a,b^	0.66
	C18:0	0.87 ^a,b^	0.92 ^A,B^	0.70	−0.93 ^A,B^	0.72
	*cis*-C18:1	0.54	0.34	0.72	−0.51	0.48
	*cis*-C18:2	0.79	0.73	0.68	−0.94 ^A,B^	0.81 ^a,b^
	C16:0	−0.14	−0.14	−0.11	−0.07	−0.03
	C20:4n6	0.21	0.04	0.44	−0.40	0.01
	C24:0	0.53	0.46	0.54	−0.23	0.17
	C24:1	0.35	0.52	0.45	−0.23	−0.07
	*cis*-C20:5n3	−0.57	−0.71	−0.53	−0.76	−0.38

Values with different lowercase superscripts show *p* < 0.05, different capital superscripts show *p* < 0.01.

## Data Availability

The mRNA-Sequencing datasets generated for this study can be found in the Sequence Read Archive (https://www.ncbi.nlm.nih.gov/sra) at NCBI, with the BioProject ID: PRJNA686190.
